# The promoting effect of byproducts from *Irpex lacteus *on subsequent enzymatic hydrolysis of bio-pretreated cornstalks

**DOI:** 10.1186/1754-6834-4-37

**Published:** 2011-10-11

**Authors:** Wanqing Du, Hongbo Yu, Lili Song, Ji Zhang, Changlong Weng, Fuying Ma, Xiaoyu Zhang

**Affiliations:** 1 School of Life Science and Technology, Huazhong University of Science and Technology, Wuhan 430074, China

## Abstract

**Background:**

*Irpex lacteus*, a versatile lignin-degrading fungus with various extracellular enzymes, has been widely used for biological pretreatment. However, most studies have focused on the change of substrate structure after biological pretreatment, and the effect of these changes on the enzymatic hydrolysis, but the effect of byproducts from biological pretreatment process on subsequent enzymatic hydrolysis is not well understood.

**Methods:**

We developed a biological pretreatment process with *I. lacteus *that can produce stimulatory byproducts that enhance the enzymatic hydrolysis of cornstalks.

**Results:**

The maximum hydrolysis yield of glucan (82%) was obtained after pretreatment for 28 days. The maximum reducing sugar yield decreased from 313.5 to 200.1 mg/g raw cornstalks after water-soluble byproducts of biological pretreatment were removed from pretreated cornstalks. The effect of byproducts on enzymatic hydrolysis was also investigated. We found that the hydrolysis efficiency of commercial cellulase preparation on cornstalks could be improved by water extracts from bio-pretreated cornstalks with hydrolytic enzyme activity and iron-reducing activity.

**Conclusion:**

The key finding suggested that byproducts from biological pretreatment play important roles in enhancing downstream hydrolysis, which might be attributable to hydrolytic enzymes and iron-reducing compounds produced by *I. lacteus*.

## Background

Low-cost and abundant agricultural residues such as cornstalks and rice straw are recognized as ideal sources of fermentable sugars for biorefinery on a sufficiently large scale [1]. However, the resistance of these residues to enzymatic hydrolysis is a major limitation for conversion of this lignocellulosic biomass to sugars [2]. Thus, pretreatment of natural lignocellulosic substrate is key to achieving high sugar yields from enzymatic hydrolysis [3,4]. The physical and chemical nature of the substrate can be altered by pretreatment processes, such as removal of lignin and hemicellulose, decreasing cellulose crystallinity and increasing surface area, resulting in improvement in hydrolysis efficiency [2,4,5].

Currently, thermochemical approaches such as dilute acid and steam-explosion pretreatment can offer high fermentable sugar yields for biorefinery, but these processes usually require high temperatures and operating pressures [5,6]. Another disadvantage of thermochemical processes is the formation of degradation byproducts such as furfural and 5-hydroxymethylfurfural during pretreatment, which inhibit the downstream enzymatic hydrolysis and fermentation [7-9]. Subsequent detoxification treatment of pretreated substrate is necessary to remove the inhibitors and enhance the bioconversion efficiency, but this increases the biorefinery cost and causes loss of soluble sugars [5,10].

Biological pretreatment processes using white-rot fungus that can degrade lignin efficiently have received considerable attention because they consume less energy and are less damaging to the environment [9]. Several white-rot fungi have been used in fungal pretreatments of agriculture residues. For example, Taniguchi *et al. *[11] reported that the susceptibility of rice straw to enzymatic hydrolysis was increased by pretreatment with *Pleurotus ostreatus*. Bak *et al. *[12] used the white-rot fungus *Phanerochaete chrysosporium *to treat rice straw by submerged fermentation (SmF), and found that from 100 g of rice straw containing 35.7 g of glucan, 20.6 g of glucose was recovered by enzymatic hydrolysis. White-rot fungal pretreatment can improve enzymatic saccharification by degrading lignin and increasing substrate porosity [11,13]; however, most biological pretreatment processes have very low hydrolysis yields [5,9], thus it is necessary to improve the process by using efficient strains that can enhance enzymatic hydrolysis.

Currently, alterations in the structure, chemistry and enzymatic hydrolysis of lignocellulose after biological pretreatment with white-rot fungi have been investigated widely, but few studies have focused on the effect on the downstream hydrolysis of byproducts from these biological pretreatments. It is usually believed that the byproducts do not inhibit the subsequent hydrolysis because biological pretreatment is a natural and mild pretreatment process [9], but it is not known whether these various byproducts such as enzymes and other metabolites, which can be left in pretreated cornstalks after the biological pretreatment, might actually enhance the downstream processes. During fungal pretreatment, efficient degradation of lignin depends mainly on the ligninolytic enzymes produced by the white-rot fungi, including lignin peroxidases [14], manganese peroxidases (Mnp) and laccases [15]. Some low-molecular-weight metabolites such as chemical oxidative agents and natural mediators of ligninolytic enzymes might be also involved in the lignin biodegradation [16,17]. In addition, white-rot fungi can produce hydrolytic enzymes that can use the substrate polysaccharides to provide the carbon source for the fungal growth. These enzymes and metabolites left in the pretreated cornstalks may continue playing an important role in the subsequent enzymatic hydrolysis. Some studies have indicated that combinations of ligninolytic enzymes, mediators and commercial cellulases might enhance the enzymatic hydrolysis of softwood [18]. Therefore, the byproducts from fungal pretreatment might contribute to the release of fermentable sugars from lignocellulose if fungal strains that can produce abundant hydrolytic and ligninolytic enzymes are used in the biological pretreatment.

The white-rot fungus *Irpex lacteus *is a versatile lignin-degrading fungus, which has been widely studied for bioremediation and food biotechnology [19]. During biodegradation, *I. lacteus *can produce various extracellular oxidative and hydrolytic enzymes [20]. Our previous research found that *I. lacteus *was able to cause efficient degradation and modification of lignin during solid-state fermentation using cornstalks as sole nutrient source, and that this could contribute to reducing the resistance of cornstalks to enzymatic hydrolysis [21]. Thus, we developed a biological pretreatment process that produced stimulatory byproducts, using *I. lacteus *to enhance the enzymatic hydrolysis of cornstalks in this study. The effect of these byproducts on the downstream hydrolysis was investigated further.

## Methods

### Microorganism and inoculum

*Irpex lacteus *CD2 was isolated (Shennong Nature Reserve, Hubei, China), and maintained on potato dextrose agar (PDA) slants at 4°. The inoculum was grown on potato dextrose broth (PDB) medium for 7 days at 28°C.

### Biological pretreatment

Cornstalks (provided by Ruyi Tian, Macheng, China) were ground to pass through a 0.9 mm screen, and then air-dried. Biological pretreatment with *I. lacteus *was carried out in 250-ml Erlenmeyer flasks with 2 g of cornstalks and 5 ml of distilled water. Flasks were sterilized in the autoclave for 30 minutes at 121°C, and aseptically inoculated with 2 ml of fungal inoculum. Cultures were statically incubated at 28°C. Samples of the pretreated cornstalks were subjected to enzymatic hydrolysis after 0, 7, 14, 28 and 42 days, respectively. Twelve flasks of cultures were collected at each pretreatment time; three of these were dried at 60°C for 72 hours to determine the weight loss and chemical components, while the remaining nine were used for enzymatic hydrolysis after biological pretreatment.

### Removal of byproducts

After pretreatment, three flasks of cultures were rinsed three times with distilled water (200 times the volume of the culture), and then subjected to subsequent enzymatic hydrolysis to evaluate the effect of the water-soluble byproducts from the biological pretreatment on the hydrolysis. In addition, three flasks of cultures were dried at 60°C for 72 hours, and then subjected to the same enzymatic hydrolysis to evaluate the effect of thermolabile byproducts on the hydrolysis.

### Enzymatic hydrolysis

The enzymatic-hydrolysis experiments were carried out using cornstalks, with 2.0% substrate in 50 mmol/l sodium acetate buffer (pH 4.8) and cellulase (30 filter paper units (FPU) per gram substrate) at 48 ± 2°C. Three parallel hydrolysis experiments were performed at each pretreatment time, using a commercial cellulase preparation (Wuxi Xuemei Enzyme Preparation Technology Co., Wuxi, China). The reaction was interrupted after 3, 6, 12, 24, 36, 48 and 72 hours of hydrolysis, and the mixture filtered through filter paper. The amounts of reducing sugar after enzymatic hydrolysis were measured with 3,5-dinitrosalicylic acid (DNS) [22], and the reducing sugar yield was calculated as follows:

$$\text{Reducing sugar yield}\left(\text{mg}∕\text{g raw cornstalks}\right)=\frac{\text{amount of reducing sugar produced after enzymatic hydrolysis}\times \text{0}\text{.9}}{\text{amount of raw cornstalks}}$$

The glucose in the hydrolyzate was also determined by a high-performance liquid chromatography (HPLC) system (model 1200, Agilent Technologies Inc., Santa Clara, US) using a column (Sugar Pak 1; Waters Ltd, Milford, US) and a refractive index (RI) detector (G1362A, Agilent Technologies Inc., Santa Clara, US). The mobile phase used was deionized water. The column was used at a flow rate of 0.6 ml/min and a column temperature of 75°C. The overall yield of glucose was calculated as follows:

$$\text{Overall}\;\text{yield}\;\text{of}\;\text{glucose}\left(\%\right)=\frac{\text{amount of glucose produced after enzymatic hydrolysis}\times \text{0}\text{.9}\times 100}{\text{amount of glucan in the raw cornstalks}}$$

The hydrolysis yield of glucan was calculated as follows:

$$\text{Hydrolysis}\;\text{yield}\;\text{of}\;\text{glucan}\left(\%\right)=\frac{\text{amount of glucose produced after enzymatic hydrolysis}\times \text{0}\text{.9}\times 100}{\text{amount of glucan in the pretreated cornstalks}}$$

### Enzymatic hydrolysis of cornstalks with byproducts

A sample (1 g) of the pretreated cornstalks was immersed in 50 ml of distilled water to obtain water extracts containing the byproducts of biological pretreatment. The extraction test was performed with shaking (150 rpm) at 28 ± 2°C for 4 hours. Samples (8 ml) of the water extracts were collected and incubated in plugged 10 ml plastic test tubes with 0.16 g of cornstalks and 30 FPU/g of commercial cellulase at 48 ± 2°C. Three parallel hydrolysis experiments were carried out at each pretreatment time. The reaction was interrupted after 3, 6, 12, 24, 36, 48 and 72 hours, and the mixture was passed through filter paper. The reducing sugar in the filtrate was measured using DNS reagent [22].

### Enzyme and iron-reducing activities

The water extracts of pretreated cornstalks were assayed to determine the ligninolytic and hydrolytic enzymes and iron-reducing activities. The activities of total cellulase, endoglucanase, cellobiohydrolase and xylanase were determined in 50 mmol/l sodium acetate buffer (pH 4.8) at 50°C by monitoring the release of reducing sugar, using the following substrates, respectively: filter paper strip (Whatman number1), carboxymethycellulose sodium, microcrystalline cellulose, and xylan [23,24]. β-glucosidase activity was determined using ρ-nitrophenyl-β-D-glucopyranoside [24], and lactase activity using 2,2-azinobis-(3-ethylbenzthiazoline-6-sulfonate) [15]. Lignin peroxidase (LiP) activity was measured by monitoring the oxidation of Azure B (pH 4.5) in 50 mmol/l sodium tartrate buffer [25]. Manganese peroxidase (Mnp) activity was measured by monitoring the formation of Mn^3+^-malonate complexes at pH 4.5 in 50 mol/l sodium malonate buffer [26]; one unit of enzyme activity was defined as the amount of enzyme releasing 1 μmol of product per minute under assay conditions. Iron-reducing activity was determined by monitoring Fe^3+^-reduction based on formation of the ferrozine-Fe^2+ ^complex, and was defined as the rate of absorbance increase at 562 nm (A/min) [27].

### Chemical components of cornstalks

The levels of acid-soluble lignin (ASL), acid-insoluble lignin (AIL), glucan and xylan in all samples were determined according to the procedures in *Determination of Structural Polysaccharides and Lignin in Biomass *(2006) from the National Renewable Energy Laboratory (NREL). Lignin content was calculated as the sum of ASL and AIL content. Xylan and glucan contents were calculated based on xylose and glucose, using anhydro corrections of 0.88 and 0.9, respectively.

## Results and discussion

### Effect of biological pretreatment on cornstalk components

**Figure 1:**
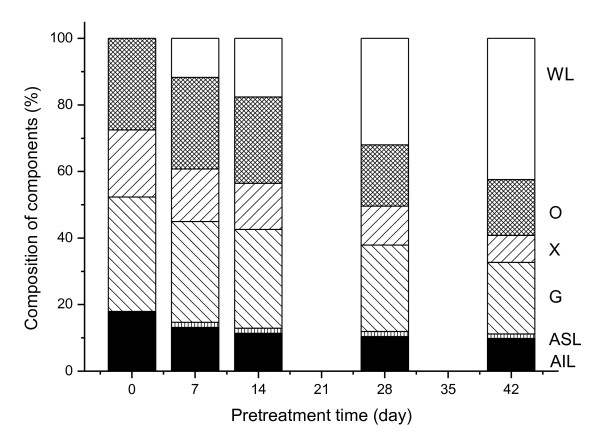
**Changes in the percentage composition of cornstalk components during the biological pretreatment**. WL = weight loss, O = other, X = xylan, G = glucan, ASL = acid-soluble lignin, AIL = acid-insoluble lignin.

We found changes in the percentage composition of cornstalk components during the biological pretreatment (Figure 1). The results indicated that *I. lacteus *was an efficient lignin-degrading fungus, able to produce a lignin-degradation yield of 37.6% after 42 days of pretreatment. During this biological pretreatment, polysaccharide degradation is unavoidable because the fungal growth requires a carbon source; the losses of glucan and xylan were 37.5% and 59.7%, respectively, after 42 days of pretreatment. The lignin and xylan degradation caused by *I. lacteus *can result in decreasing the recalcitrance of the cornstalks to enzymatic hydrolysis, but the glucan degradation increases the glucose loss during the pretreatment, therefore it is important to achieve a balance between the increase in overall saccharification yield and the consumption of polysaccharides during the biological pretreatment. Interestingly, the removal of lignin and xylan was more rapid at the early stages of pretreatment than at the advanced stages, with losses of 28%, 31.5% and 13.7%s for lignin, xylan and glucan, respectively, after 14 days of pretreatment. From this, it can be concluded that *I. lacteus *utilizes xylose more easily than glucose as its carbon source at the early stage of pretreatment, which results in the decrease in glucose consumption.

### Enzymatic hydrolysis of cornstalks

**Figure 2:**
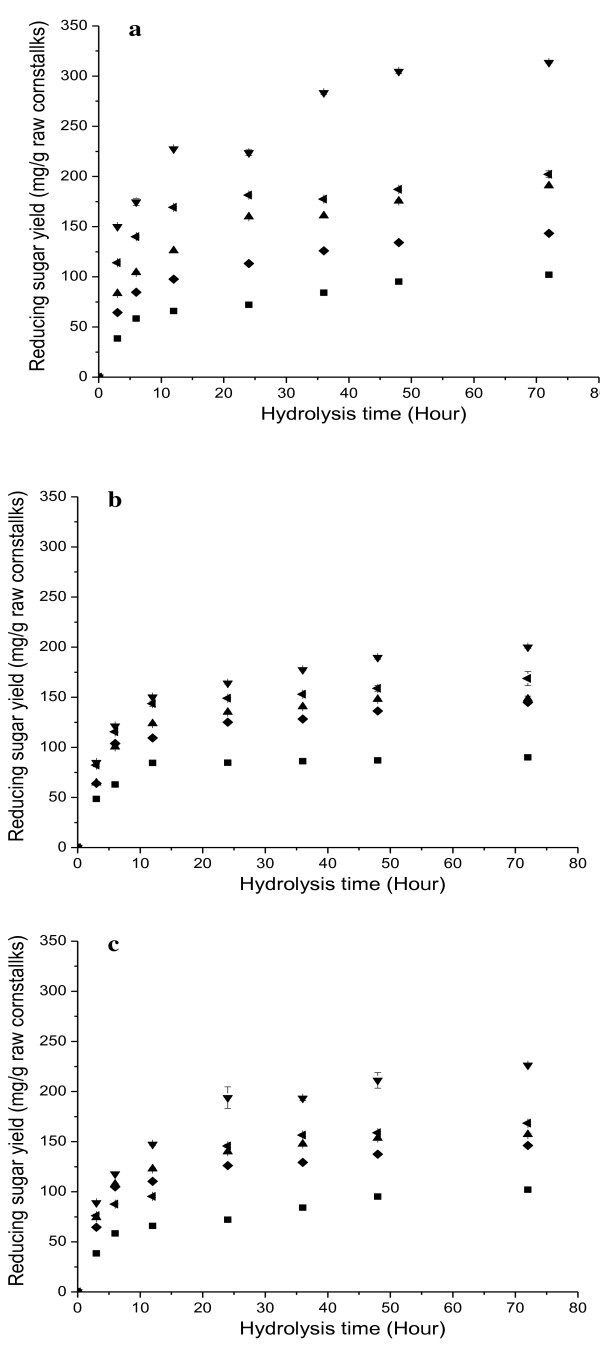
**Time courses of reducing sugar yields (mg/g raw cornstalks) during enzymatic hydrolysis of cornstalks the biological pretreatment with *Irpex lacteus *for 0 (■), 7 (♦), 14 (▲), 28 (▼) and 42 (◀) days with 30 FPU/g enzyme loading**. **(A) **Direct enzymatic hydrolysis; **(B) **enzymatic hydrolysis after removal of water-soluble byproducts; **(C) **enzymatic hydrolysis after heat treatment.

**Figure 3:**
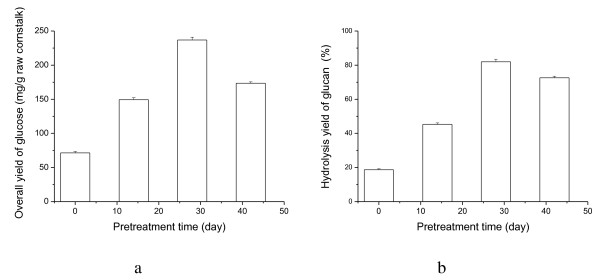
**Overall yield of glucose (mg/g raw cornstalk) and hydrolysis yield of glucan (%) after 72 hours of enzymatic hydrolysis of cornstalks pretreated with *Irpex lacteus *for 0, 14, 28 and 42 days with 30 FPU/g enzyme loading**.

*I. lacteus *significantly improved the enzymatic hydrolysis of cornstalks (Figure 2a, 3a). The maximum reducing sugar yield (313.5 mg/g cornstalk) and overall glucose yield (236.9 mg/g raw cornstalks) was achieved after 28 days of pretreatment, increasing by 2.07 and 2.32 times, respectively, compared with untreated cornstalks. Hydrolysis of pretreated straw has been reported widely [28]. The hydrolysis yields of straw pretreated with thermochemical processes are usually about 80 to 100%, but those of straw pretreated with biological processes are usually very low [5]. The maximum hydrolysis yield of cornstalk glucan was 82.0% of theoretical yield after 28 days of biological pretreatment in the present study (Figure 3b), which was much higher than the maximum glucan hydrolysis yield (64.9%) reported for rice straw pretreated with SmF using *P. chrysosporium *in a recent study [12], and only slightly lower than that of cornstalk pretreated with thermochemical processes. In the present study, we used solid-state fermentation (SSF) without an additional nutrient source during the biological pretreatment. SSF simulates the natural growth state of fungi, and is particularly suitable for deconstruction of lignocellulose by white-rot fungi. Compared with SmF, SSF has many potential advantages for biological pretreatment, including lower energy and water consumption, lower cost, simpler fermentation technique, and lower risk of contamination [9,29]. Thus, SSF using *I. lacteus *is a promising process for biological pretreatment of cornstalks.

Despite the relatively high hydrolysis yield from the biological pretreatment, the process still needs a long pretreatment time because of the restrictions imposed by the growth and degradation rates of the fungi. Recently, some combined processes using thermochemical and biological pretreatment have been used to speed up the biological processes. In our previous studies, the time taken for the biological process could be shortened to 15 days when fungal pretreatment with *I. lacteus *was combined with alkali pretreatment [30]. Future development of powerful biotechnology and engineering tools will undoubtedly speed up this process further. Nevertheless, biological pretreatment usually requires a longer time than thermochemical pretreatment. For example, the time taken for dilute acid pretreatment is usually shorter than 60 minutes [4,5]; however, special anticorrosion heating equipment with compression resistance and high water consumption are required for this. By contrast, a biological pretreatment process using SSF is carried out at near room temperature (20-30°C), has relatively low energy and water consumption, and does not need special equipments, which can partly compensate for the production cost involved with the longer pretreatment time. It is noteworthy that the biological process needs no chemicals and is thus less damaging to the environment, which can save the cost of pollution treatment. In addition, it was reported previously that *I. lacteus *is able to grow and reproduce efficiently under non-sterile conditions, indicating that an on-farm biological pretreatment process might be developed in future [19]. Having the production process on the farm may offer lower cost, and practical and sustainable processes. Thus, biological pretreatment using SSF should be a competitive process compared with thermochemical processes, both economically and from an environmental protection point of view.

Enhancement of enzymatic hydrolysis after biological pretreatment has usually been attributed to the lignin biodegradation [5,11]. Selective lignin-degrading fungi are usually considered good candidates for biological pretreatment because they can significantly decrease the lignin content in lignocellulose. Although *I. lacteus *is an efficient lignin-degrading fungus, we found that it had a selective lignin-degrading ability only in the early stages of pretreatment. *I. lacteus *produced only slight degradation of the lignin from 14 to 28 days of biological pretreatment (Figure 1). However, it was found that the enzymatic hydrolysis of pretreated cornstalks increased greatly during this period. The reducing sugar yield of the pretreated cornstalk after 28 days of pretreatment was 1.70 time that after 14 days of pretreatment, indicating that there might be other factors playing important roles in enhancing the enzymatic hydrolysis.

### Effect of byproducts on the enzymatic hydrolysis

It is well known that white-rot fungi can produce abundant ligninolytic and hydrolytic enzymes during SSF [29]. The enzymes left in bio-pretreated cornstalks can act as byproducts and might affect the subsequent enzymatic hydrolysis. We found that the reducing sugar yields of pretreated cornstalks decreased significantly after water-soluble byproducts including enzymes and other metabolites were removed from the cornstalks (Figure 2b). The greatest effect of this removal on the enzymatic hydrolysis of cornstalk was seen after 28 days of pretreatment; the reducing sugar yield after the removal of byproducts was 200.1 mg/g raw cornstalk, which was only 61.5% of that achieved before the removal of the byproducts.

**Figure 4:**
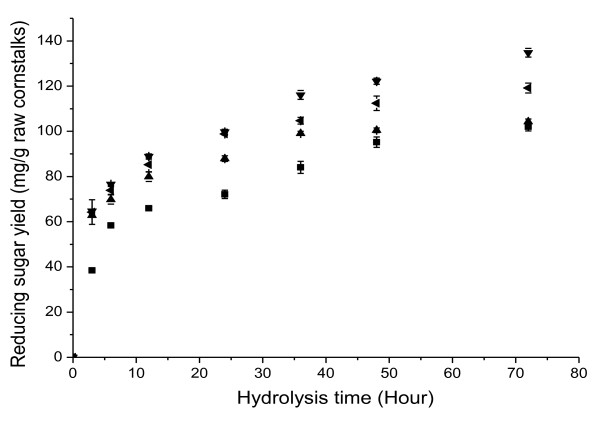
**Time courses of reducing sugar yields (mg/g raw cornstalks) during enzymatic hydrolysis of raw cornstalks with commercial cellulase preparations (30 FPU/g) and water extract of cornstalks pretreated with *Irpex lacteus *for 14(▲), 28(▼) and 42(◀) days**. The enzymatic hydrolysis without water extract was used as control (■).

To evaluate further the effect of these byproducts on enzymatic hydrolysis, cornstalks were hydrolyzed using a commercial cellulase preparation and water extract of bio-pretreated cornstalks. The hydrolysis efficiency of cellulase on the cornstalks was improved by addition of the water extract containing byproducts (Figure 4), with reducing sugar yields after enzymatic hydrolysis with water extract being higher than after enzymatic hydrolysis without water extract (*P *< 0.05). The water extract of cornstalks pretreated for 28 days enhanced enzymatic hydrolysis more significantly than other extracts, and the reducing sugar yield increased 32.1% compared with the control. Therefore, the improvement in enzymatic hydrolysis after biological pretreatment with *I. lacteus *resulted not only from the alteration in the structure of cornstalks, as with thermochemical pretreatments, but also from the synergy between these byproducts and the cellulase preparation on the subsequent enzymatic hydrolysis.

Enzymes in biological byproducts are usually thermolabile. We found that heat treatment of pretreated cornstalks inactivated the enzymes and decreased the enzymatic hydrolysis significantly, showing that the enzymes in the byproducts were the main factors enhancing the subsequent hydrolysis (Figure 2c). However, the reducing sugar yields after heat treatment were higher than those after removal of the water-soluble byproducts. The reducing sugar yield of cornstalks pretreated for 28 days was 226.3 mg/g after heat treatment, which was 1.13 times greater than that after removal of the water-soluble byproducts, indicating that in addition to enzymes, some thermostable constituents produced by *I. lacteus *might play a role in the enzymatic hydrolysis.

### Enzyme and iron-reducing activities

Cellulase and xylanase activities were detectable in water extracts of pretreated cornstalks (Table 1), indicating that some hydrolytic enzymes remained in the cornstalks after biological pretreatment. Some studies have reported that cellulases produced by *I. lacteus *and *Aspergillus niger *have a synergistic effect on the hydrolysis of cellulose [31]. Thus, the cellulase and xylanase remaining in the cornstalks might contribute to the improvement of subsequent enzymatic hydrolysis. Interestingly, we did not find any activity of Lac, LiP or Mnp in the water extracts of pretreated cornstalks. Our previous study also found no ligninolytic activity during the early stage of SSF with *I. lacteus*, and only slight LiP activity in the late stage of SSF. However, this result did not indicate that *I. lacteus *does not produce the ligninolytic enzyme during SSF, because the ligninolytic enzyme produced by *I. lacteus *can attach to the cell wall of mycelia so that its extraction by water is difficult [32]. The enzymes attaching to mycelia might promote the subsequent enzymatic hydrolysis hydrolysis by removing lignin during this process, but further study is necessary to confirm this assumption.

**Table 1:** Specific enzyme activities in the biological pretreated cornstalks

Pretreatment length, days	FPA, FPU/g	EG, IU/g	CBH, IU/g	BG, IU/g	XYL, IU/g	FeRA, A/min
14	1.08 ± 0.03	1.35 ± 0.02	2.72 ± 0.05	0.17 ± 0.01	4.41 ± 0.17	0.17 ± 0.01
28	1.03 ± 0.02	1.42 ± 0.16	2.31 ± 0.05	0.19 ± 0.01	7.40 ± 0.07	0.66 ± 0.02
42	1.28 ± 0.06	1.65 ± 0.11	2.64 ± 0.17	0.12 ± 0.01	8.33 ± 0.20	0.30 ± 0.01

BG = β-glucosidase activity; CBH = cellobiohydrolase; EG = endoglucanase; FeRA = iron-reducing activity; FPU = filter paper unit; XYL = xylanase

We were able to detect high iron-reducing activity in the water extracts from pretreated cornstalks, indicating that the byproducts of biological pretreatment included some Fe^3+ ^reductants. The highest iron-reducing activity was found in the extract after 28 days of pretreatment. Fe^3+ ^reductants play important roles in driving the Fenton reaction, in which Fe^2+ ^and H_2_O_2 _react to form hydroxyl radicals. Generation of hydroxyl radicals is considered to be related to the lignin degradation and cellulose depolymerization during fungal biodegradation [19,33]. During the subsequent enzymatic hydrolysis of cornstalks, the reductants produced by *I. lacteus *may act as an indirect source of hydroxyl radicals, resulting in the improvement in enzymatic hydrolysis through oxidation of lignin and depolymerization of cellulose. It is believed that iron-reducing activity is induced by some putative iron reductases. However, we found that some low-molecular-weight metabolites which are not iron reductases, such as aromatic carboxylic acids produced by white-rot fungi, might be involved in the reduction of Fe^3+^[27]. These metabolites might be thermostable constituents in the byproducts, which enhance the enzymatic hydrolysis.

## Conclusion

*Irpex lacteus *is a promising white-rot fungus for biological pretreatment of cornstalks. The maximum glucan hydrolysis yield of 82% can be obtained after 28 days of biological pretreatment. The byproducts from biological pretreatment play important roles in enhancing the subsequent enzymatic hydrolysis. The removal of water-soluble byproducts from pretreated cornstalks can result in a large decrease in the subsequent enzymatic hydrolysis required. The promoting effect of byproducts on the hydrolysis may contribute to the level of hydrolytic enzymes and iron-reducing compounds left in the cornstalks after biological pretreatment.

## Competing interests

The authors declare that they have no competing interests.

## Authors' contributions

WD designed and carried out experiments, analyzed the results and wrote the manuscript. HY and XZ analyzed the results, and revised the manuscript. LS and JZ coordinated the full study. CW and FM helped to analyze data, and participated in the manuscript editing. All authors read and approved the final manuscript.
